# Characterization of human breast tissue microbiota from core needle biopsies through the analysis of multi hypervariable 16S-rRNA gene regions

**DOI:** 10.1038/s41598-018-35329-z

**Published:** 2018-11-15

**Authors:** Lara Costantini, Stefano Magno, Davide Albanese, Claudio Donati, Romina Molinari, Alessio Filippone, Riccardo Masetti, Nicolò Merendino

**Affiliations:** 1 Department of Ecological and Biological Sciences (DEB), Tuscia University, Largo dell’Università, 01100 Viterbo, Italy; 2 Fondazione Policlinico Universitario A.Gemelli IRCCS, Largo Agostino Gemelli 8, 00168 Rome, Italy; 3 Computational Biology Unit, Research and Innovation Centre, Fondazione Edmund Mach, Via E. Mach 1, 38010 S. Michele all’Adige, Italy; 4 Università Cattolica del Sacro Cuore, Largo Agostino Gemelli 8, 00168 Rome, Italy

## Abstract

Breast microbiota compositions are not well understood, and a few recent reports have begun to explore the correlation between breast tissue dysbiosis and cancer. Given that various methods for breast microbiota detection were used, the aim of the present paper was to clarify which hypervariable region of the 16S-rRNA gene (V2, V3, V4, V6 + 7, V8, and V9) is the most informative for breast tissue microbiota. Core needle biopsies (CNBs) were compared with surgical excision biopsies (SEBs) to find a less invasive form of recovery useful for the analysis of a larger statistical population and potentially for diagnostic use of breast tissue microbiota. Finally, this study was the first to analyse the breast microbiota of tumours and paired normal tissues of a Mediterranean population. Our findings showed that the V3 region is the most informative for breast tissue microbiota, accounting for 45% of all reads. No significant differences were found between CNB and SEB specimens in terms of total reads and numbers of Operational Taxonomic Units (OTUs). Moreover, we find that more similarities than differences exist between tumours and adjacent normal tissues. Finally, the presence of the *Ralstonia* genus is associated with breast tissue.

## Introduction

Breast cancer remains the most common tumour found in women worldwide. Despite the significant therapeutic advances made, European breast cancer mortality predictions are estimated at 92,700 deaths for 2018 and are anticipated to rise to 93,300 by 2020^1,2^. While the aetiology of this malignancy remains unclear, like other cancers, it is a multifactorial disease that likely emerges from a combination of genetic and non-hereditary factors. Among non-hereditary factors, microbiota are attracting considerable interest^3^. It is estimated that 15% to 20% of cancer cases are driven by microbial pathogens and that even more malignancies are associated with an altered composition of microorganisms that colonize human tissues, referred to as dysbiosis^4^. Studies based on gnotobiotic animal models support the presence of oncogenic activity related to dysbiosis, proving the cancerous or tumour suppressive activities of certain bacterial strains^5,6^. Nevertheless, most of these studies are associative, and as such, they cannot determine whether dysbiosis is a cause or consequence of the onset of the disease. For this reason, the causal relationship between microbiota and cancer remains unclear.

In addition to exploring bacterial entry from the nipple (from the skin, from lactation, and from sexual contact)^7,8^, some studies have proposed the presence of a gut-breast axis along which bacteria present in the maternal gut may reach the mammary gland through an endogenous route involving dendritic cells^9^. Moreover, further studies show that mammary dysbiosis may lead to the formation of lactational mastitis, an inflammation of mammary gland in which opportunistic pathogenic bacteria outgrow healthy commensal bacteria^10^. Evidence shows that the treatment of lactational mastitis with orally administered probiotics recovered from human milk is more effective than antibiotic and then it supports the presence of a gut-breast axis^11^. However, breast microbiota compositions are not well understood, and only recently have works begun to explore the correlation between breast tissue dysbiosis and cancer. These works agree that breast tissues have unique microbiota distinct from other body sites and characterized by a predominance of *Proteobacteria* as the most abundant phylum followed by *Firmicutes*^12,13^. However, to date, it is not clear if a difference between tumour and paired normal tissues within the breast does exist^12,14^. In addition, existing studies on breast tissue microbiota have applied different detection methods, and it is thus difficult to compare them. Recent evidence shows that the microbiota of normal tissues are more similar to those of adjacent tumour tissues than to the microbiota of breast tissues of women without cancer^15^. This phenomenon may be interpreted in two different ways: dysbiosis is antecedent to carcinogenic events and can establish a microenvironment prone to cancer or there is no correlation between the two events. However, knowledge of the microbiota of breast cancer patients remains in its infancy. Given this context, further studies must be conducted to understand which of the two possibilities occurs in cases of breast cancer.

Considering that breast tissue microbiota have been poorly characterized and with several methods, the first aim of the present work was to clarify which hypervariable region of the 16S-rRNA gene is the most descriptive for breast tissue microbiota by returning the highest number of sequenced reads and by detecting phyla identified in previous works^12,13^. The most informative region was in turn used for breast tissue characterization. In addition, with a view to the diagnostic use of microbiota, we compared the bacterial content of tissues derived from surgical excision biopsies (SEBs) using a less invasive recovery technique involving core needle biopsies (CNBs) drawn from patients with diagnosed with breast cancer. For this purpose, fresh tissue samples of both cancer and paired healthy tissues were collected from CNBs and SEBs to evaluate if the former has the same resolving power as the most invasive SEB procedure. Finally, we for the first time analysed the breast microbiota of tumour and paired normal tissues of a Mediterranean population.

## Results

### Hypervariable region selection

The first taxonomic identification of 32 collected specimens (six samples from patients that underwent both SEB and CNB procedures were discarded from CNB group) was carried out on reads obtained via the amplicon sequencing of seven hypervariable regions of the 16S-rRNA gene (V2, V3, V4, V6 + V7, V8, and V9). Total read counts drawn from the CNB (median 475,337) and SEB (median 609,335) sampling methods were not significantly different (*p* = 0.33). As was expected, amplicons ranged in size from 200 to 300 bp with a mean read length of 213.22 ± 21.07 bp.

The environmental control samples collected to identify potential contaminations involved due to specimen exposure to the external environment contained an undetectable amount of microbial gDNA.

**Figure 1:**
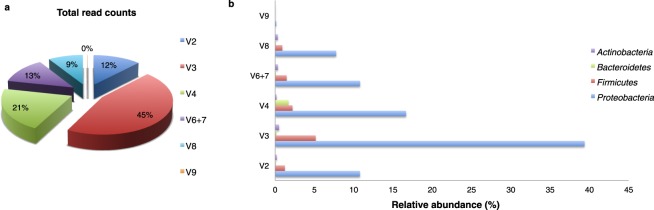
Comparison of the efficiency of analysed hypervariable regions of the 16S-rRNA gene (n = 32). (**a**) Distribution of obtained total read counts for each analysed hypervariable region. (**b**) Evaluation of read distributions of the analysed 16S-rRNA gene hypervariable regions and of each phylum found to compare the informative power of the regions. In both panels values are presented as relative abundances as percentages of total read counts obtained by combining data drawn for all of the analysed specimens (both SEBs and CNBs, healthy and cancerous).

An analysis of the amplified regions shows that the percentage of reads was not homogeneous across the hypervariable regions. Indeed, 45% of the reads were drawn from the V3 region followed for the V4, V6 + V7, V2, and V8 regions (21%, 13%, 12%, and 9% of the reads, respectively). The V9 region was the less informative, contributing less than 0.1% of the sequencing reads (Fig. 1, panel a). Similar proportions of mapped reads were also found from sampling and tissue type groups (see Supplementary Fig. S1). The percentage of each found phylum was evaluated for each hypervariable region (Fig. 1, panel b). The presence of four major phyla (*Proteobacteria*, *Firmicutes*, *Actinobacteria*, and *Bacteroidetes*) was supported by all of the analysed hypervariable regions (Fig. 1, panel b), and the estimated proportions of each phylum were found to be comparable. We thus obtained comparable estimates of sample biodiversity from the different hypervariable regions. Across the regions, the *Proteobacteria* phylum was the most abundant followed by *Firmicutes*, *Actinobacteria*, and *Bacteroidetes*, respectively. Indeed, 85% of the reads mapped onto the V3 region were classified as belonging to phylum *Proteobacteria*, 11% belonged to *Firmicutes*, and the rest belonged to *Actinobacteria* and *Bacteroidetes* (Fig. 1, panel b). Likewise, for all reads belonging to the V4 region, the region with the second most reads, 83% of reads were classified as deriving from the *Proteobacteria* phylum, 11% derived from *Firmicutes*, and the rest derived from *Bacteroidetes* and *Actinobacteria* (Fig. 1, panel b). The only difference found between the V3 and V4 regions concerning phyla proportions was an enhanced detection of *Actinobacteria* for the V3 region against an increased level of *Bacteroidetes* found for the V4 region, although this involved only 0.5% and 1.6% of all reads, respectively (Fig. 1, panel b). Completely different results were found for the V9 region, which presented lower levels of biodiversity with only taxa of the *Proteobacteria* phylum (Fig. 1, panel b).

### Microbiota analysis

**Figure 2:**
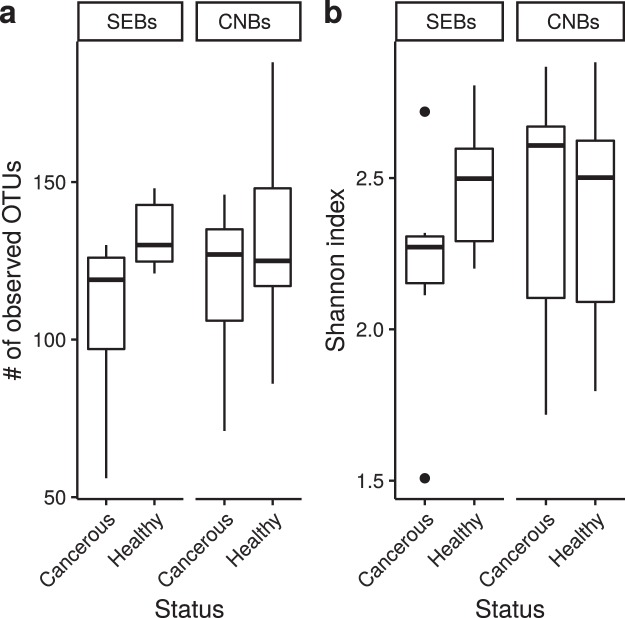
Alpha-diversity analysis after rarefaction (n = 30). A comparison of the two alpha-diversity measures for the two tissue and sampling types: **(a)** number of observed OTUs, **(b)** Shannon index.

From the analysis of the most informative hypervariable region we used one region to conduct our microbiota analysis to avoid systematic biases^16^. Therefore, the V3 region was selected given that it was the region to which the largest number of reads (45%) belonged (Fig. 1, panel a). A dataset of 12,668,490 reads was generated with a mean coverage of 70,223.42 reads per sample. Sampling heterogeneity was reduced by rarefaction (without replacements) to 4500 reads per sample. One tumour SEB sample with less than 4500 reads and the paired normal SEB sample were discarded. Six samples from patients that underwent both SEB and CNB procedures were discarded from CNB group. Breast microbiota of the paired normal and tumour tissues of 9 patients undergoing CNBs and of 6 patients subjected to SEBs were surveyed for a total of 30 specimens. After rarefaction, a total amount of 738 operational taxonomic units (OTUs) was obtained based on 97% sequence similarity (see Supplementary Dataset S1). The number of observed OTUs was not significantly different among healthy tissues and paired tumour tissues of SEBs (medians: 130 for healthy tissues and 119 for cancerous tissues), and of CNBs (medians: 125 for healthy tissues and 127 for cancerous tissues) (*p* = 0.0684 and *p* = 0.0922 SEBs and CNBs, respectively, generalized linear model^17^, quasi-Poisson families), revealing no significant difference in richness between the two classes of samples (Fig. 2, panel a; Supplementary Dataset S2). Similarly, between subjects, the number of observed OTUs was not significantly different among SEBs and CNBs of healthy and cancerous tissues (*p* = 0.816 and *p* = 0.434, for healthy and cancerous tissues respectively, generalized linear model, Poisson family). The differences were not found to be significant when applying Shannon entropy (*p* = 0.69 and p = 0.379 for healthy and cancerous tissues, respectively, generalized linear model, Gaussian family).

**Figure 3:**
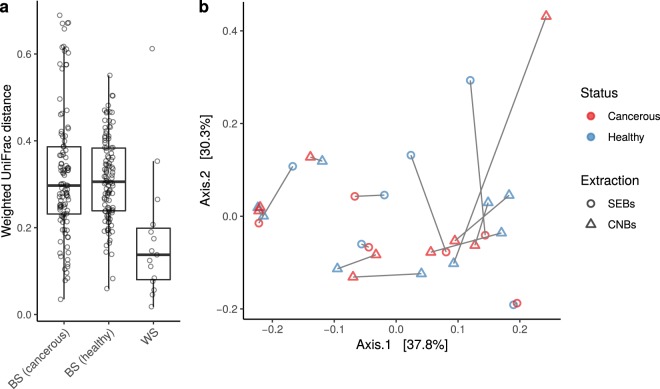
Dissimilarity analysis (beta-diversity) of the healthy and cancerous CNB and SEB samples. (n = 30). (**a**) The weighted UniFrac distance was used to analyse distances between cancerous and healthy samples of different subjects (between subjects, BS) in comparison to healthy and cancerous samples of the same subject (within subject, WS). **(b)** Principal coordinate analysis (PCoA) based on Weighted UniFrac distances.

**Figure 4:**
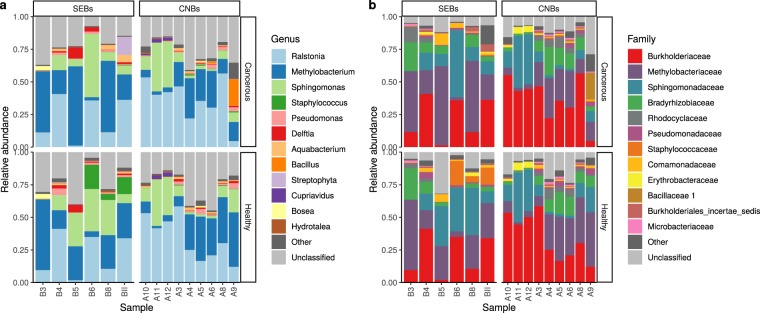
Relative abundances measured at the genus and family levels. Barplots of the taxonomic profiles with each bar representing a subject and with each coloured box showing a bacterial taxon. **(a)** Barplots of each specimen measured at the genus level; **(b)** barplots of each specimen measured at the family level.

The Shannon diversity index did not show significant differences between healthy-adjacent and tumour tissues for both CNBs and SEBs (*p* = 0.959 and *p* = 0.0547, respectively, linear model, Gaussian family) (Fig. 2, panel b). Weighted UniFrac distances were calculated to investigate dissimilarities (beta-diversity) between the SEB and CNB samples of both healthy-adjacent and cancer tissues. The survey shows that differences found within subjects (i.e., distances between healthy-adjacent and cancerous samples for each subject) were significantly less pronounced than those found between subjects for both healthy-adjacent (*p* = 6.78 × 10^−5^) and cancerous tissues (*p* = 2.02 × 10^−4^, Wilcoxon rank-sum test, FDR corrected *p* values - Fig. 3, panel a). Moreover, no significant differences were found when comparing all healthy-adjacent tissues to all cancerous tissues (*p* = 0.84). In support of this, a principal coordinate analysis (PCoA) of weighted UniFrac distances showed no clear divisions between tumour and healthy-adjacent tissues (Fig. 3, panel b). PCoA (using weighted UniFrac distances) was also used to highlight differences correlated with histoprognostic grade and molecular subtypes of tissue samples (see Supplementary Fig. S2). However, even in this case, no clear segregation of samples was found. Finally, taxonomic identification at the family/genus level for each individual patient was carried out to better understand differences found in the examined population (Fig. 4). Confirming the previous taxonomic analysis, OTUs found in the V3 region belonged to the four different phyla (*Proteobacteria*, *Firmicutes*, *Bacteroidetes*, and *Actinobacteria*). As is shown in Fig. 4, roughly 50–75% of relative abundances belonged to *Ralstonia*, *Methylobacterium*, and *Sphingomonas* genera (see Supplementary Dataset S3). The remaining 25–50% were mainly constituted by *Staphylococcus* and *Pseudomonas* genera and by *Bradyrhizobiaceae* and *Rhodocyclaceae* families (see Supplementary Dataset S3 and S4). To confirm the beta-diversity analysis shown in Fig. 3, the relative abundance of cancerous and healthy-adjacent tissues drawn from the same patient was found to be essentially the same while major differences were found among different subjects. Nevertheless, in some cases differences were also found in the same patient. For example, cancerous samples B5, and B8 showed an increase in the relative abundance of *Methylobacterium* and a decrease in the relative abundance of *Sphingomonas* in comparison to their healthy counterparts. Similarly, cancerous samples A5, A6, and A8 showed a decrease in the relative abundance of *Methylobacterium* concomitant with an increase in the *Ralstonia* genus relative to the healthy controls. These differences were not found in the other subjects.

## Discussion

While the presence of specific microbiota in human milk has been known for several years^18^, only in the last few years breast tissue microbiota have been evaluated irrespective of lactation and in correlation with cancer^12–15,19–21^. Such studies have been made possible with the use of next-generation sequencing (NGS) technologies, which allow for the simultaneous analysis of large volumes of genetic material drawn from uncultivable bacteria in cost-effective and time-saving modalities^22^. However, considerable differences in methodologies used, e.g., specimen treatment after collection, gDNA isolation, target hypervariable region selection for sequencing, and sequence analysis workflows, have resulted in considerable heterogeneity in results, delaying the assessment of the existence of a link between dysbiosis and breast cancer. For example, there is a lack of consensus regarding which hypervariable region to amplify for breast tissue microbiota description. It is know that specific hypervariable regions are more likely to identify certain taxa^23^ and that because different microenvironments are colonized by different microbial populations, the amplification of a hypervariable region solely due to its specific use for another biological niche is inappropriate. To date there is no evidence regarding which hypervariable region is the most representative for describing breast tissue microbiota. Such an assessment appears fundamental to the correct characterization of this new microenvironment.

To shed light on this issue, we for the first time simultaneously analysed seven hypervariable regions of the 16S-rRNA gene (V2, V3, V4, V6 + V7, V8, and V9) to identify the hypervariable region producing the most reads in the same workflow. We found the V3 region to be the most representative in terms of read counts, accounting for 45% of all reads, while the V8 and V9 regions contribute to the fewest reads. According to these data, even though regions V4 and V6 + V7 are the most informative for gut microbiota description^24^, the use of the same regions to describe breast tissue microbiota could underestimate the totality of the analysed population^12,14,19,20^. Instead, the strategy adopted in the most recent studies of Hieken *et al*., 2016, Wang *et al*., 2017, and Thompson *et al*., 2017 using primers that cover the entire V3-V5 region appears to be the best strategy as confirmed by the present study, from which we found the V3 and V4 regions to identify 45% and 21% of examined reads, respectively^13,15,21^. Moreover, this choice may overcome minor differences that we found between the identification of *Actinobacteria* and *Bacteroidetes* from the V4 and V3 regions (Fig. 1, panel b). In support of this, a study comparing several hypervariable regions to describe oral microbiota shows that it is important to use the V1-V3 and V7-V9 regions to obtain results similar to those of 16S-rRNA gene Sanger sequencing^25^. Nevertheless, even if the use of multiple hypervariable regions seems to be the best strategy for describing a complex community, to the best of our knowledge no method yet combines OTUs across multiple variable regions^26^. In summary, V3 region sequencing can provide a fairly complete account of breast tissue microbiota and especially when the amplification of small regions of DNA is desirable. In other cases, the sequencing of an amplicon covering both V3 and V4 regions is more appropriate at higher resolutions^27^. Finally, in light of the present findings, future applications of the same analysis even for the V1 and V5 regions could help to complete our overview and eventually confirm that the V3 region is the most informative of breast tissue microbiota.

More detailed analyses of the specimens considered in this study were carried out on the V3 region. Both our multiple hypervariable region evaluation and V3 analysis show that breast tissue microbiota do not have comparable compositions to those of other body sites (e.g., the gut, skin, vagina, oral cavity, and bladder)^28–32^. Indeed, in this tissue niche *Proteobacteria* constitute the most abundant phylum followed by *Firmicutes*, *Actinobacteria*, and *Bacteroidetes* in descending order (Fig. 1, panel b). On the other hand, we find evident similarities with human milk for which *Proteobacteria* is also the most abundant phylum followed by the other phyla (*Firmicutes*, *Actinobacteria*, and *Bacteroidetes*, respectively)^33^. These findings complement those of previous studies conducted on breast tissue microbiota^12,13,19^.

The second aim of the present study was to compare samples drawn from CNBs with those obtained from conventional surgical biopsies, SEBs, to determine if the former can apply the same level of resolving power as the most invasive surgical procedure. The procedure from which CNBs can be obtained allows for a more limited contamination of specimens even if the tissue levels are lower than those of SEBs. Indeed, even though breast tissues drawn from SEBs were obtained under fully aseptic conditions, surgical excision involves an unavoidable exposure to the external environment^34,35^. However, in the present study, the collection of environmental controls showed that such external contamination was undetectable.

Our findings show that no significant differences in total read counts and number of OTUs were found between our CNB and SEB specimens, thus exhibiting their equal capacity to describe breast tissue microbiota. Then, since CNBs are obtained from a less invasive procedure, this may be used as a predictive tool for the evaluation of potential dysbiosis in concomitance with the determination of histoprognostic grade and hormone receptor status even in a screening population study^36^.

Finally, the ultimate goal of the present study was to evaluate if differences exist between cancerous and paired normal tissues drawn from CNB and SEB specimens. We first found no differences in the number of OTUs in normal tissues than in paired tumour tissues for both CNBs and SEBs, suggesting a same level of richness between tumour tissues and healthy-adjacent tissues (Fig. 2, panel a). Similarly, our biodiversity analysis of alpha- and beta-diversity levels did not reveal significant differences between cancerous and paired normal tissues (Fig. 2 panel b; Fig. 3). However, differences between healthy and cancerous tissues drawn from the same patient in comparison to differences between healthy and cancerous tissues drawn from all of the analysed patients were found to be significant, indicating that there are more differences between subjects than between healthy and cancerous tissues collected from the same patient (Fig. 3, panel a). The fact that bacterial diversity within samples varies most between individuals was also found by Urbaniak *et al*., 2014^12^. The observation that more similarities than differences exist between tumour and adjacent normal tissues is in agreement with the results of Wang *et al*., 2017 who found that invasive carcinoma and paired normal tissues show no major shifts in overall diversity or microbiomic content^15^. Similar results were found by Urbaniak *et al*., 2016 who found normal tissues from women with benign tumours to be more similar to normal adjacent tissues from cancer patients than to normal tissues from healthy women^19^. Given that previous studies have highlighted differences between normal tissues of cancer and non-cancer patients^12,15,19^ and between nipple aspirate fluids in women with or without a history of breast cancer^20^, the presence of similar microbiota in cancerous and paired normal tissues could indicate a predisposition to carcinogenesis as previously suggested^15^. Applying such results to a larger population could thus involve using the microbiota analysis of breast tissues obtained with CNB as a predictive tool for measuring breast cancer risk.

In support of variability found between individuals, relative abundance measured at the family/genus level for each individual patient was assayed (Fig. 4). We are the first to find that the presence of genus *Ralstonia*, *Proteobacteria* phylum, is the most abundant genus found in breast tissue while the presence of this genus had previously only been related to human milk^7^. This finding may be attributed to procedures used for microbial gDNA isolation and analysis, which likely allow for a greater recovery of useful gDNA and for its more accurate analysis. Indeed, samples were obtained under aseptic conditions and were freshly processed to limit contamination and genetic material loss as is found in frozen and formalin-fixed paraffin-embedded tissues^37^. Moreover, most gDNA from living microbes was isolated, removing non-target DNA as free floating and human DNA^38^. Finally, the most representative hypervariable region of the 16S-rRNA gene V3 was used to perform a metagenomic analysis. In turn, these procedures likely allowed us to secure a better description of the target microbial population. In addition, in this study, breast tissue microbiota of the Italian population (with geographic, racial, and dietary differences compared to previously analysed populations) were analysed for the first time^39^.

It is noteworthy that among this population, three patients showed a decrease in the relative abundance of *Methylobacterium* concomitant with an increase in the *Ralstonia* genus. However, the only correlation between *Ralstonia* dysbiosis and cancer cited in the literature is related to gastric cancer^40^ while a correlation with breast cancer has never been investigated.

Following *Ralstonia* in terms of abundance, the presence of *Methylobacterium* and *Sphingomonas* genera in *Proteobacteria* phylum was confirmed consistent with Xuan *et al*., 2014, Urbaniak *et al*., 2014, and Wang *et al*., 2017^12,14,15^. In two patients we also found differences between paired normal and cancerous tissues where the relative abundance of *Methylobaterium* in cancerous tissues increases while the relative abundance of *Sphingomonas* decreased in comparison to the healthy counterparts as shown in prior studies^12,14,15^. These differences were not found in all of the analysed patients and for this reason they are not deemed statistically significant.

Given strong inter-individual variations found in breast bacterial compositions, it could be assumed that breast tissue microbiota can fall into distinct group types and that the dysbiosis of these different taxa inevitably spurs a different interplay of polymicrobial interactions that in correlation with genetic predispositions and/or hormone receptor statuses could drive the establishment of a carcinogenic event.

This study is limited by the small sample size used and as discussed above through our exclusion of V1 and V5 regions from the analysis. Future studies of larger samples must be conducted to confirm with confidence a correlation between microbiota and breast cancer with reference to a positive control of normal tissue drawn from healthy individuals.

Nevertheless, the present study showed that the V3 hypervariable region of the 16S-rRNA gene among the other six analysed regions (V2, V4, V6 + V7, V8, and V9) presents the largest number of mapped reads between the CNB and SEB samples. Percutaneous breast biopsies (CNBs) appear to be reliable and safe clinical tools for sufficiently obtaining (even if with a smaller tissue samples) thorough microbiota descriptions of breast tissues comparable to those generated from more invasive procedures such as surgical biopsies (SEBs). Finally, similar microbiota were found in cancerous and surrounding normal tissues, suggesting that when dysbiosis occurs, it is an antecedent to a carcinogenic event and may establish a microenvironment prone to cancer. In any case, breast microbiota and their correlations with cancer remain unclear, and further studies of larger populations must be conducted to understand this relationship. Given this context, as CNBs are less invasive than SEBs, and the form could allow for the collection of a larger number of samples as was done through the Human Microbiome Project; this approach could be used to help clarify whether breast tissue dysbiosis may contribute to carcinogenesis. Following such clarifications and confirmations, CNB may prove useful as a predictive tool for the dysbiosis evaluation of the healthy population.

## Methods

### Ethics statement and patient enrolment

CNBs and SEBs of fresh breast tissues were obtained from patients diagnosed with breast cancer undergoing surgical treatment at the Fondazione Policlinico Universitario Agostino Gemelli IRCCS in Rome, Italy. Ethical approval for this study was obtained from the ethics committee of the Fondazione Policlinico Universitario Agostino Gemelli IRCCS (approval number: 8407/18 ID: 1915) and all methods were performed in accordance with all relevant guidelines and regulations. Patients provided written informed consent for sample collection and subsequent analysis. Clinical and biological patient data (age, BMI, menopausal status, menarche advent, number of pregnancies, comorbidity, family history of breast cancer, hormone therapy compliance, histoprognostic grade, hormone receptor status (Estrogen Receptor, ER; Progesterone Receptor, PR; Human Epidermal Growth Factor Receptor 2, HER2; Ki-67)) and molecular subtypes were collected and are listed in Table 1. Patients eligible to participate in the study were females of over 18 years of age with tumours larger than or equal to 2 cm in size who had not been subjected to any current pharmacological cancer treatments and who had no active clinical breast infections or necrotic tissues.

**Table 1:** Characteristics of and clinical data on the volunteers.

	CNBs	SEBs	Total
Number of patients	9	7	16
Age – average (range)	55 (46–71)	65 (46–82)	59 (46–82)
BMI – average (range)	24.6 (18.8–28.2)	27.8 (20.8–36.5)	26.0 (18.8–36.5)
Pre-menopausal – n (%)	1 (11.1%)	1 (14.3%)	2 (12.5%)
Peri-menopausal – n (%)	2 (22.2%)	1 (14.3%)	3 (18.8%)
Post-menopausal – n (%)	6 (66.7%)	5 (71.4%)	11 (68.8%)
Menarche – average (range)	13.1 (11–17)	12.1 (10–16)	12.7 (10–17)
Pregnancy – n (%)	8 (88.9%)	7 (100%)	15 (93.8%)
Number of pregnancies – average (range)	1.7 (0–4)	2.3 (2–4)	2.1 (0–4)
Comorbidity – n (%)	4 (44.4%)	5 (71.4%)	9 (56.3%)
Family history of breast cancer – n (%)	3 (33.3%)	2 (28.6%)	5 (31.3%)
Hormone therapy – n (%)	1 (11.1%)	2 (28.6%)	3 (18.8%)
Grade G1– n (%)	0 (0.0%)	1 (14.3%)	1 (6.3%)
Grade G2 – n (%)	8 (88.9%)	3 (42.9%)	11 (68.8%)
Grade G3 – n (%)	1 (11.1%)	3 (42.9%)	4 (25.0%)
Estrogen Receptor positive (ER+) – n (%)	8 (88.9%)	6 (85.7%)	14 (87.5%)
Estrogen Receptor negative (ER−) – n (%)	1 (11.1%)	1 (14.3%)	2 (12.5%)
Progesterone Receptor positive (PR+) – n (%)	8 (88.9%)	6 (85.7%)	14 (87.5%)
Progesterone Receptor negative (PR−) – n (%)	1 (11.1%)	1 (14.3%)	2 (12.5%)
Human Epidermal Growth Factor Receptor 2 positive (HER2+) – n (%)	1 (11.1%)	0 (00.0%)	1 (6.2%)
Human Epidermal Growth Factor Receptor 2 negative (HER2−) – n (%)	8 (88.9%)	7 (100%)	15 (93.8%)
High Ki-67 (H-Ki-67) – n (%)	8 (88.9%)	5 (71.4%)	13 (81.2%)
Low Ki-67 (L-Ki-67) – n (%)	1 (11.1%)	2 (28.6%)	3 (18.8%)
Luminal A (ER+/PR+/HER2−/ L-Ki-67) – n (%)	1 (11.1%)	2 (28.6%)	3 (18.8%)
Luminal B/ HER2- (ER+/PR+/HER2+) – n (%)	7 (77.8%)	4 (57.1%)	11 (68.8%)
HER2-like (ER−/PR−/HER2+) – n (%)	1 (11.1%)	0 (00.0%)	1 (6.2%)
Triple negative (ER−/PR−/HER2−) – n (%)	0 (0.0%)	1 (14.3%)	1 (6.2%)

Data are expressed as the means with ranges shown in brackets (i.e., min and max values) or as the number of subjects (shown in brackets) of the relative percentage of subjects.

High Ki-67: positive cells to Mib1 antibody ≥20%; Low Ki-67: positive cells to Mib1 antibody <20%.

CNBs: core needle biopsies; SEBs: surgical excision biopsies; BMI: body mass index.

### Tissue collection and processing

CNB and SEB specimens were collected from 16 patients (12 for CNBs and 7 for SEBs, three patients underwent both procedures). For both samples (i.e., CNBs and SEBs), both tumour and healthy adjacent tissues were collected for a total of 38 specimens. For CNBs, two imaging-guided sampling approaches involving the use of two different sterile semiautomatic devices equipped with 14-gauge needles were applied to tumour and paired normal tissues after skin surface disinfection with povidone-iodine (Betadine). For tumour SEBs, a slice of each tumour mass radial section was collected under sterile conditions after tumour masses were excised (i.e., neoplastic tissue surrounded by a marginal zone) from the patients in an operating room under aseptic conditions. To avoid cross-contamination, a different disposable sterile scalpel from that used for excision was used to obtain the tumour section. For both procedures, healthy adjacent tissue was collected from outside of the marginal zone positioned approximately 5 cm away from the tumour’s edge. After both excisions were made, fresh tissues (approximately 15 mg and 200 mg for CNBs and SEBs, respectively) were immediately placed in sterile study code-labelled DNA-free vials on ice and were processed within 3 hours of collection. As an environmental control, for both surgical procedures, a tube filled with 200 μL of TSB buffer (Ultra-Deep Microbiome Prep, Molzym, Bremen, Germany) was left open until an excision was made and it was then processed as the tissues specimens were. Both CNB and SEB fresh tissue samples were cut into small pieces (less than 0.2 × 0.2 cm) with a sterile scalpel under sterile conditions. Immediately thereafter, microbial genomic DNA (gDNA) isolation was performed.

### Microbial gDNA isolation

To remove non-target DNA, an Ultra-Deep Microbiome Prep kit (Molzym, Bremen, Germany) was used according to the manufacturer’s protocol. This allowed us to degrade free floating and human DNA and to isolate gDNA from living microbes in fresh clinical samples. All extractions were performed with the same kit lot, in a designated clean pre-PCR area using DNA-free reagents and consumables. The concentration of the obtained microbial gDNA samples was estimated using a Qubit 3.0 Fluorometer (Life Technologies, Carlsbad, CA, USA) and Qubit dsDNA HS Assay Kit (Life Technologies, Carlsbad, CA, USA). The gDNA samples were then stored at −20 °C until further use.

### Preparation of samples and sequencing of 16S-rRNA gene amplicons

gDNA drawn from each clinical sample was amplified using an Ion 16S Metagenomic Kit (Life Technologies, Carlsbad, CA, USA). Using this kit we amplified seven of nine hypervariable regions of the 16S-rRNA gene through two sets of primers: one set for the V2, V4, and V8 regions and the other for the V3, V6 + V7, and V9 regions. The PCR was performed by preparing two reactions for each clinical sample for each of the two primer sets using a PCR Sprint thermocycler (Thermo Hybaid, Ashford, UK) following the kit manufacturer’s instructions. Amplification products of each set were then pooled in equimolar amounts and the final mixtures were purified using magnetic beads of the Agencourt AMPure XP Reagent (Beckman Coulter, Beverly, MA, USA). We then used 2 μL of each mixture to calculate the DNA input for library preparation using the Qubit dsDNA HS Assay Kit (Life Technologies, Carlsbad, CA, USA). Concentrations of 10–100 ng in 79 μL of dilution buffer were used to prepare libraries using the Ion Plus Fragment Library Kit (Life Technologies, Carlsbad, CA, USA). This allowed us to first execute end-repair procedures and then to ligate adapters after purification and perform nick-repairs. As different clinical samples were combined into pooled libraries, barcoded adapters were employed via the Ion Xpress Barcode Adapters Kit (Life Technologies, Carlsbad, CA, USA). After an additional purification step, 50 ng of the obtained library was amplified to enhance the presence of amplicons with heterogeneous adapters (P1-X, rather than P1-P1 or X-X) following the kit manufacturer’s instructions (Ion Plus Fragment Library Kit, Life Technologies, Carlsbad, CA, USA). After another purification step, each library was quantified using the Qubit dsDNA HS Assay Kit (Life Technologies, Carlsbad, CA, USA) and was pooled to the correct dilution. For template preparation on Ion Sphere Particles (ISPs), pooled libraries were subjected to emulsion PCR performed using the Ion PGM Hi-Q View OT2 Kit (Life Technologies, Carlsbad, CA, USA) and the Ion One Touch 2 System instrument (Life Technologies, Carlsbad, CA, USA). Only template-positive ISPs were then selected with Ion PGM Enrichment beads (Life Technologies, Carlsbad, CA, USA) using the Ion One Touch ES instrument (Life Technologies, Carlsbad, CA, USA). Finally, positive ISPs for all of the samples and controls were randomly loaded onto six different Ion 316 Chips v2 BC (Life Technologies, Carlsbad, CA, USA) and were sequenced with an Ion PGM Sequencer using an Ion PGM Hi-Q View Sequencing Kit (Life Technologies, Carlsbad, CA, USA).

### Sequence analysis

BAM files obtained from Ion PGM Sequencer output were directly processed with Ion Reporter Software 5.6 (Life Technologies, Carlsbad, CA, USA). Within this programme, the 16S Metagenomic workflow works with MicroSEQ(R) 16S Reference Library v2013.1 and Greengenes v13.5 databases to align and obtain a taxonomic identification of sequences. Raw data were first analysed with Ion Reporter Software 5.6 (Life Technologies, Carlsbad, CA, USA) to obtain information on relative taxonomic abundances from primers. Statistical significance between read counts of the different sampling types was calculated through a *t*-student test.

OTU analyses were then performed on hypervariable region V3, as this region generated the most reads. Raw reads were pre-processed using micca^41^, which makes extensive use of VSEARCH^42^ for OTU clustering and sequence manipulation. Raw V3 sequences were extracted using the micca trim command with primers 5′-ACTCCTACGGGAGGCAGCAG-3′ and 5′-CCAGCAGCCGCGGTAATAC-3′. Sequences were truncated at 240 bp and reads with an expected error rate^43^ of greater than 0.5% were discarded. Sequences were clustered (applying the *de novo* greedy algorithm available through micca) at 97% identity and chimeric OTUs were discarded. Taxonomy was assigned to OTUs using the RDP classifier^44^ (version 2.11) wrapper. Multiple sequence alignment was performed using the Nearest Alignment Space Termination^45^ (NAST) algorithm and a phylogenetic tree was inferred from the FastTree wrapper available through micca. Alpha diversity was modelled with generalized linear models through the R stats package. P-values were not corrected for multiple testing.

## Electronic supplementary material

Supplementary Figures

Dataset 1, Dataset 2, Dataset 3, Dataset 4

## Data Availability

Datasets generated through the current study are available from the corresponding author on reasonable request.

## References

[1] Malvezzi M, Carioli G, Bertuccio P, Boffetta P, Levi F, La Vecchia C, Negri E (2018). European cancer mortality predictions for the year 2018 with focus on colorectal cancer. Annals of Oncology.

[2] Carioli G (2017). Trends and predictions to 2020 in breast cancer mortality in Europe. The Breast.

[3] Francescone R, Hou V, Grivennikov SI (2014). Microbiome, Inflammation, and Cancer. Cancer J..

[4] Bhatt AP, Redinbo MR, Bultman SJ (2017). The role of the microbiome in cancer development and therapy: Microbiome and Cancer. CA Cancer J. Clin..

[5] Arthur JC (2012). Intestinal inflammation targets cancer-inducing activity of the microbiota. Science..

[6] Donohoe DR (2014). A gnotobiotic mouse model demonstrates that dietary fiber protects against colorectal tumorigenesis in a microbiota- and butyrate-dependent manner. Cancer Discov..

[7] Hunt KM (2011). Characterization of the diversity and temporal stability of bacterial communities in human milk. PLos One.

[8] Cabrera-Rubio R (2012). The human milk microbiome changes over lactation and is shaped by maternal weight and mode of delivery. Am. J. Clin. Nutr..

[9] Rescigno M (2001). Dendritic cells express tight junction proteins and penetrate gut epithelial monolayers to sample bacteria. Nat. Immunol..

[10] Patel, S. H. *et al*. Culture independent assessment of human milk microbial community in lactational mastitis. *Sci. Rep*. **7** (2017). 10.1038/s41598-017-08451-7 PMC5552812 28798374

[11] Arroyo R (2010). Treatment of Infectious Mastitis during Lactation: Antibiotics versus Oral Administration of Lactobacilli Isolated from Breast Milk. Clin. Infect. Dis..

[12] Urbaniak C (2014). Microbiota of Human Breast Tissue. Appl. Environ. Microbiol..

[13] Hieken, T. J. *et al*. The Microbiome of Aseptically Collected Human Breast Tissue in Benign and Malignant Disease. *Sci. Rep*. **6** (2016). 10.1038/srep30751 PMC4971513 27485780

[14] Xuan C (2014). Microbial Dysbiosis Is Associated with Human Breast Cancer. PLoS ONE.

[15] Wang, H. *et al*. Breast tissue, oral and urinary microbiomes in breast cancer. *Oncotarget* **8** (2017). 10.18632/oncotarget.21490 PMC5675698 29152146

[16] Lozupone CA (2013). Meta-analyses of studies of the human microbiota. Genome Res..

[17] Bolker BM (2009). Generalized linear mixed models: a practical guide for ecology and evolution. Trends Ecol. Evol..

[18] Fernández L (2013). The human milk microbiota: Origin and potential roles in health and disease. Pharmacol. Res..

[19] Urbaniak C (2016). The Microbiota of Breast Tissue and Its Association with Breast Cancer. Appl. Environ. Microbiol..

[20] Chan, A. A. *et al*. Characterization of the microbiome of nipple aspirate fluid of breast cancer survivors. *Sci. Rep*. **6** (2016). 10.1038/srep28061 PMC4914981 27324944

[21] Thompson KJ (2017). A comprehensive analysis of breast cancer microbiota and host gene expression. PLOS ONE.

[22] Metzker ML (2010). Sequencing technologies - the next generation. Nat. Rev. Genet..

[23] He Y (2013). Comparison of microbial diversity determined with the same variable tag sequence extracted from two different PCR amplicons. BMC Microbiol..

[24] Aloisio I (2016). Evaluation of the effects of intrapartum antibiotic prophylaxis on newborn intestinal microbiota using a sequencing approach targeted to multi hypervariable 16S rDNA regions. Appl. Microbiol. Biotechnol..

[25] Kumar PS, Brooker MR, Dowd SE, Camerlengo T (2011). Target Region Selection Is a Critical Determinant of Community Fingerprints Generated by 16S Pyrosequencing. PLoS ONE.

[26] Barb JJ (2016). Development of an Analysis Pipeline Characterizing Multiple Hypervariable Regions of 16S rRNA Using Mock Samples. PLOS ONE.

[27] Takahashi S, Tomita J, Nishioka K, Hisada T, Nishijima M (2014). Development of a Prokaryotic Universal Primer for Simultaneous Analysis of Bacteria and Archaea Using Next-Generation Sequencing. PLoS ONE.

[28] Huttenhower C (2012). Structure, function and diversity of the healthy human microbiome. Nature.

[29] Grice EA, Segre JA (2011). The skin microbiome. Nat. Rev. Microbiol..

[30] Aagaard K (2012). A Metagenomic Approach to Characterization of the Vaginal Microbiome Signature in Pregnancy. PLoS ONE.

[31] Dewhirst FE (2010). The Human Oral Microbiome. J. Bacteriol..

[32] Hilt EE (2014). Urine Is Not Sterile: Use of Enhanced Urine Culture Techniques To Detect Resident Bacterial Flora in the Adult Female Bladder. J. Clin. Microbiol..

[33] Urbaniak, C., Angelini, M., Gloor, G. B. & Reid, G. Human milk microbiota profiles in relation to birthing method, gestation and infant gender. *Microbiome* **4** (2016). 10.1186/s40168-015-0145-y PMC4702315 26739322

[34] Paajanen H, Hermunen H (2009). Does Preoperative Core Needle Biopsy Increase Surgical Site Infections in Breast Cancer Surgery? Randomized Study of Antibiotic Prophylaxis. Surg. Infect..

[35] Al-Hilli Z, Thomsen KM, Habermann EB, Jakub JW, Boughey JC (2015). Reoperation for Complications after Lumpectomy and Mastectomy for Breast Cancer from the 2012 National Surgical Quality Improvement Program (ACS-NSQIP). Ann. Surg. Oncol..

[36] Waaijer L (2015). Impact of preoperative evaluation of tumour grade by core needle biopsy on clinical risk assessment and patient selection for adjuvant systemic treatment in breast cancer: Preoperative grading and clinical risk assessment in breast cancer. Br. J. Surg..

[37] Ananian V (2011). Tumoural specimens for forensic purposes: comparison of genetic alterations in frozen and formalin-fixed paraffin-embedded tissues. Int. J. Legal Med..

[38] Handschur M, Karlic H, Hertel C, Pfeilstöcker M, Haslberger AG (2009). Preanalytic removal of human DNA eliminates false signals in general 16S rDNA PCR monitoring of bacterial pathogens in blood. Comp. Immunol. Microbiol. Infect. Dis..

[39] Fallani M (2010). Intestinal Microbiota of 6-week-old Infants Across Europe: Geographic Influence Beyond Delivery Mode, Breast-feeding, and Antibiotics. J. Pediatr. Gastroenterol. Nutr..

[40] Tseng, C.-H. *et al*. Gastric microbiota and predicted gene functions are altered after subtotal gastrectomy in patients with gastric cancer. *Sci. Rep*. **6** (2016). 10.1038/srep20701 PMC4748256 26860194

[41] Albanese, D., Fontana, P., De Filippo, C., Cavalieri, D. & Donati, C. MICCA: a complete and accurate software for taxonomic profiling of metagenomic data. *Sci. Rep*. **5** (2015). 10.1038/srep09743 PMC4649890 25988396

[42] Rognes T, Flouri T, Nichols B, Quince C, Mahé F (2016). VSEARCH: a versatile open source tool for metagenomics. PeerJ.

[43] Edgar RC, Flyvbjerg H (2015). Error filtering, pair assembly and error correction for next-generation sequencing reads. Bioinformatics.

[44] Wang Q, Garrity GM, Tiedje JM, Cole JR (2007). Naive Bayesian Classifier for Rapid Assignment of rRNA Sequences into the New Bacterial Taxonomy. Appl. Environ. Microbiol..

[45] De Santis TZ (2006). NAST: a multiple sequence alignment server for comparative analysis of 16S rRNA genes. Nucleic Acids Res..

