# Supplementary material for: Characterization of human breast tissue microbiota from core needle biopsies through the analysis of multi hypervariable 16S-rRNA gene regions
Source: Sci Rep. 2018 Nov 15;8:16893. doi: 10.1038/s41598-018-35329-z (PMC6237987; doi:10.1038/s41598-018-35329-z)

Supplementary Information

Supplementary Figures

Characterization of human breast tissue microbiota from core needle biopsies through the analysis of multi hypervariable 16S-rRNA gene regions

Lara Costantini*, Stefano Magno, Davide Albanese, Claudio Donati, Romina Molinari, Alessio Filippone, Riccardo Masetti, Nicolò Merendino

Supplementary Figure S1. Total reads count for surgical excision biopsies (SEBs) and core needle biopsies (CNBs) from healthy-adjacent tissues and cancerous tissues.

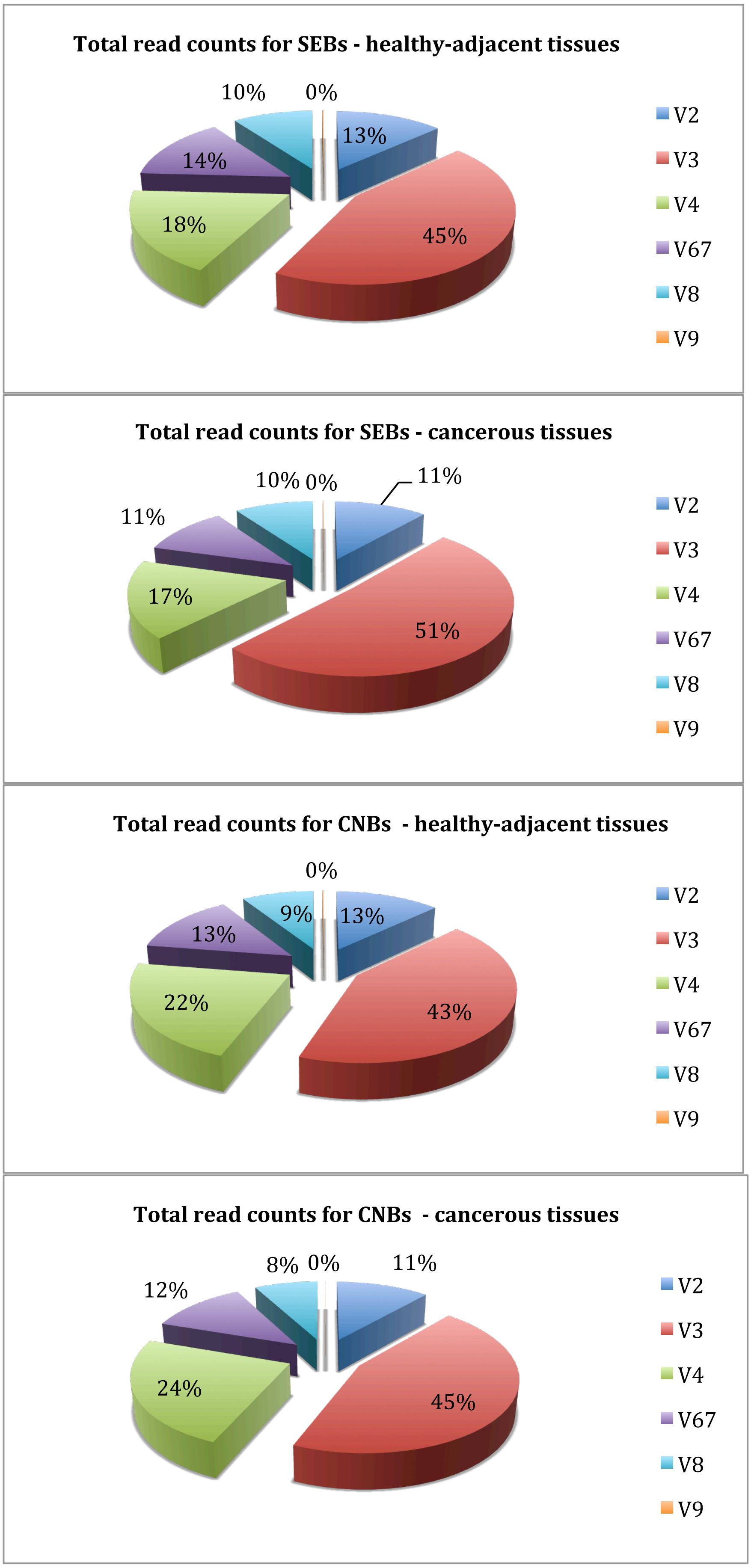

Supplementary Figure S2. Dissimilarity analysis (beta-diversity) between healthy and cancerous CNB and SEB samples for histoprognostic grade and hormone receptor status groups (n=30). Weighted UniFrac distance, using principal coordinate analysis (PCoA), was used to analyse total data distribution along the axes for histoprognostic grade and hormone receptor status groups.

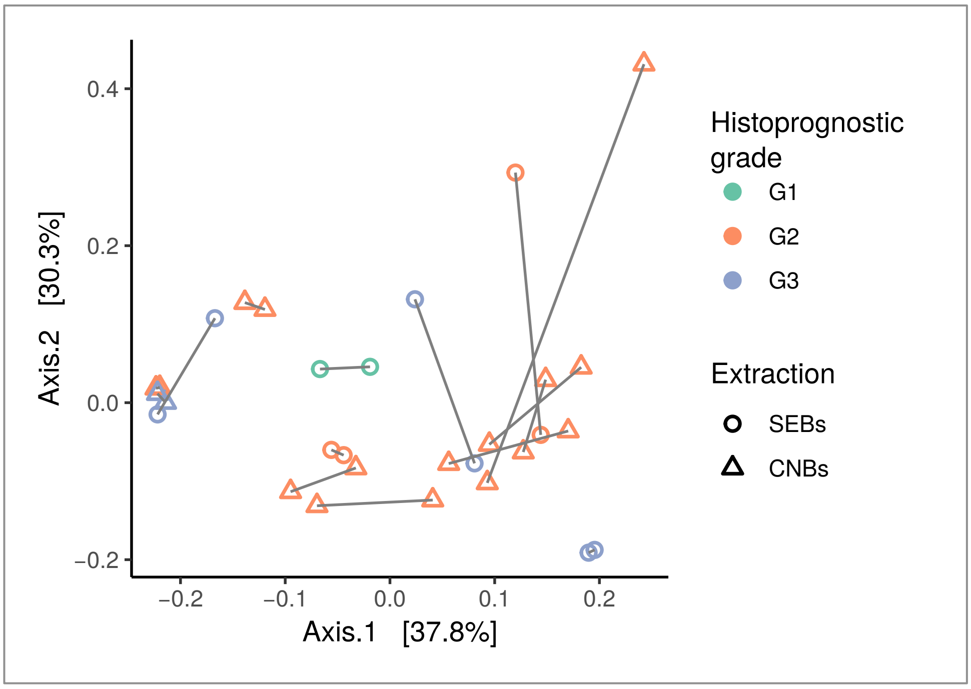

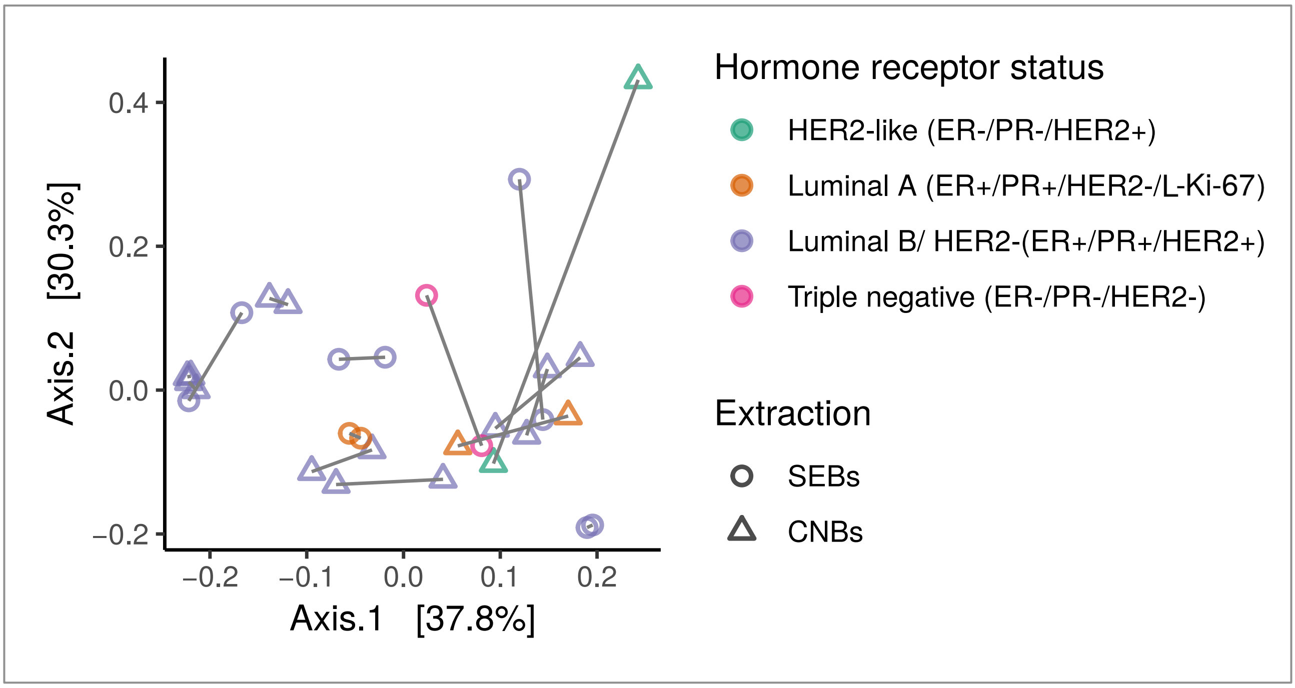

Supplement: Supplementary file 1 — Supplementary Figures [file 41598_2018_35329_MOESM1_ESM.pdf]
